# Potential protective effect against SARS-CoV-2 infection by *APOE* rs7412 polymorphism

**DOI:** 10.1038/s41598-022-10923-4

**Published:** 2022-05-04

**Authors:** Isabel Espinosa-Salinas, Gonzalo Colmenarejo, Cristina M. Fernández-Díaz, Marta Gómez de Cedrón, J. Alfredo Martinez, Guillermo Reglero, Ana Ramírez de Molina

**Affiliations:** 1 IMDEA-Food Institute, CEI UAM+CSIC, 28049 Madrid, Spain; 2 Center for Nutrition Research (CIN), Navarra Institute for Health Research (IdiSNA), 31008 Pamplona, Spain; 3 Center of Biomedical Research in Physiopathology of Obesity and Nutrition (CIBEROBN), Institute of Health Carlos III, 28029 Madrid, Spain; 4 Institute of Food Science Research (CIAL), CEI UAM+CSIC, 28049 Madrid, Spain

**Keywords:** Preventive medicine, Risk factors

## Abstract

The pandemic burden caused by the SARS-CoV-2 coronavirus constitutes a global public health emergency. Increasing understanding about predisposing factors to infection and severity is now a priority. Genetic, metabolic, and environmental factors can play a crucial role in the course and clinical outcome of COVID-19. We aimed to investigate the putative relationship between genetic factors associated to obesity, metabolism and lifestyle, and the presence and severity of SARS-CoV-2 infection. A total of 249 volunteers (178 women and 71 men, with mean and ± SD age of 49 ± 11 years) characterized for dietary, lifestyle habits and anthropometry, were studied for presence and severity of COVID-19 infection, and genotyped for 26 genetic variants related to obesity, lipid profile, inflammation, and biorhythm patterns. A statistically significant association was found concerning a protective effect of *APOE* rs7412 against SARS-CoV-2 infection (p = 0.039; OR 0.216; CI 0.084, 0.557) after correction for multiple comparisons. This protective effect was also ascribed to the APOɛ2 allele (p = 0.001; OR 0.207; CI 0.0796, 0.538). The genetic variant rs7412 resulting in ApoE2, genetic determinant of lipid and lipoprotein levels, could play a significant role protecting against SARS-CoV-2 infection.

## Introduction

COVID-19 pandemic that we are currently experiencing caused by SARS-CoV-2 has affected nearly 350 million people and caused almost 5.6 million deaths globally as of January 2022^1^. Impaired immune response or exacerbated pro-inflammatory response are associated to the main complications of people affected of COVID-19. In addition, emerging evidence demonstrates close link between metabolism and the viral disease process^2^.

The clinical course of COVID-19 is highly variable. Elderly or patients with some comorbidities such as diabetes mellitus, hypertension, renal dysfunction, cardiovascular diseases (CVDs) and cancer are the most vulnerable groups to suffer severe or fatal cases due to this infection^3–5^.

Effective control of metabolic parameters (control of glucose, lipid levels, blood pressure and chronic inflammation) might represent a successful approach to dealing with the acute effects of infection by decreasing virus entry into cells and reducing local inflammatory response. Studies have also shown that the nutritional status (determined by Nutritional Risk Tools, NRT) of old patients with COVID-19 has a significant impact on their clinical outcomes^6^.

Recently, the importance of genotype-environment interactions in shaping the different metabolic phenotypes has been stressed, where diet, exercise, circadian rhythms and gut microbiome appear as the most important environmental factors^7,8^. The metabolic variability among individuals in the response to diet should be taken into consideration to individualize healthy eating advice, which will promote a reduction in the risk of non-communicable diseases (NCDs). Genetic factors are unmodifiable, but their effects can be modulated through lifestyle and environmental factors^6^.

COVID-19’s aetiology (its cause or set of causes), beyond SARS-CoV-2 infection, is currently the subject of a large research effort worldwide, given that the disease, at least in its more severe manifestations, occurs through a host huge inflammatory reaction. In this way, several studies have suggested associations between the lipid profile and the infection status or severity of this disease^9–11^. These studies disclose a severe deregulation of lipoproteins leading to increased triglycerides (TG), together with an alteration of lipoprotein particle levels and an increase in VLDL subclasses with intermediate size^9^. In turn, other studies showed that COVID-19 adult patients presented lower levels of high-density lipoprotein (HDL-C), which was correlated with a higher risk of developing severe events. In addition, increased TG/HDL-C ratio was associated with disease severity and mortality in COVID-19 patients^10,11^. Therefore, the current state of research reflects a promising scenario for the discovery of useful metabolism-markers for the early identification of patients at high risk of infection and serious diagnosis.

Likewise, an involvement of circadian rhythms regarding the regulation of the immune system as well as in the severity of infections has been observed^12,13^. Although the knowledge concerning the mechanisms of interaction at a molecular level of the biological clock and viral infections is still scarce, it is known that viruses are likely to alter the biological processes of infected cells in order to promote their replication and to spread to various tissues. Possible deregulations of circadian rhythms could have a substantial impact on the severity of the symptoms^14^.

In this perspective, Precision Nutrition is a complementary therapeutic strategy in the management of metabolic and immune alterations associated to COVID-19 poor prognosis. Personalized nutrition combines different aspects to obtain a holistic view of the individual, such as nutrigenetics, detailed phenotyping, and a big set of data obtained from omics technologies (metabolomics, metagenomics, transcriptomics, lipidomics…). Importantly, nutritional interventions based on omics knowledge represents an effective tool to fight against infections and chronic conditions. Hence, Precision Nutrition should also be considered as a complementary tool within the COVID-19 pandemic^15^.

Although COVID-19 is not primarily a metabolic disease, proper control of some metabolic parameters such as glycemia, blood pressure and lipid levels represent a key therapeutic approach to prevent and ameliorate the acute effects of this viral infection. Within the framework of Precision Nutrition, herein we have analyzed 26 genetic variants in metabolic genes related to obesity, inflammation, chronobiology and nutrient sensing to evaluate their putative associations to the susceptibility and evolution of symptomatology to SARS- CoV2 infection. We identify minor allele rs7412 polymorphism in *APOE* gene is associated to diminished susceptibility to SARSCoV-2 infection.

## Results

### Descriptive analysis of COVID-19 disease in the GENYAL platform

A study to identify single nucleotide genetic polymorphisms involved in the SARS-CoV-2 infection was conducted in the GENYAL population. A total of 371 volunteers from this cohort answered the COVID-19 questionnaire designed for this study. COVID-19 questionnaire plus genotyping data were finally restricted to a total of 249 subjects with enough reliable data (178 women and 71 men, with mean and ± SD age of 49 ± 11 years) presented in Table 1. Of the total sample, 210 subjects were considered young adults (< 60 years old, mean age of 46 ± 10 years) while 39 subjects were senior adults (≥ 60 years old; 65 ± 3 years). Table 1 shows the descriptive data of the study sample categorized by gender, age, and infection status (“infected”: 50; “not infected”: 141 subjects; and volunteers with suspected SARS-CoV-2 infection without diagnosis, labeled "not clear": 58). Considering the potential impact of comorbidities on SARS-CoV-2 susceptibility and outcome of SARS-CoV-2 infection, possible associations between comorbidities and SARS-CoV-2 infection status were tested and the results of the analysis are presented in Table 1. No associations were found between SARS-CoV-2 infection status and the studied comorbidities, with the exception of immunosuppression. The data according to the severity of the symptoms (for those infected) are shown in Table S1. No significant associations were found between the studied SNPs involved in lipid metabolism, obesity, inflammation and biorhythms, and COVID-19 severity. Likewise, no positive association was found between SARS-CoV-2 infection and the lifestyle parameters analyzed.

**Table 1:** Descriptive phenotypic and clinical data of the study sample categorized by gender, age and SARS-CoV-2 infection.

Variables	Total % (*n* = 249)	Male (*n* = 71)	Female (*n* = 178)	*p*	Young Adults (Age ≤ 60) (n = 210)	Older Adults (Age ≥ 60) (n = 39)	*p*	Virus Infection (*n* = 50)	Not Clear (*n* = 58)	No Infection (*n* = 141)	*p*
**Age**	49.13 ± 11.23	47 ± 12	50 ± 11	0.030	-	-	-	45.0 ± 9.0	50.91 ± 11.1	49.85 ± 11.7	0.003
**BMI (kg/m**^**2**^**)**	27.44 ± 5.09	29.0 ± 4.7	26.8 ± 5.1	0.002	27.37 ± 5.1	27.86 ± 5.1	0.582	28.26 ± 5.84	26.55 ± 4.34	27.52 ± 5.07	0.350
**WC (cm)**	91.47 ± 15.2	100.4 ± 15.2	88.1 ± 13.8	< 0,001	90.95 ± 15.1	94.18 ± 15.4	0.232	94.79 ± 16.92	90.36 ± 13.47	90.83 ± 15.24	0.379
**Obesity**											
No	68.02	67.61	70.79	0.648	70.00	69.23	1	62.00	68,97	73,05	0,350
Yes	31.98	32.39	29.21		30.00	30.77		38.00	31,03	26,95	
**Hypertension**											
No	86.44	81.69	92.70	0.020	90.48	84.61	0.262	88.89	82,76	92,91	0,098
Yes	13.56	18.31	7.30		9.52	15.38		11.11	17,24	7,09	
**Diabetes**											
No	96.79	97.18	96.63	1	97.14	94.87	0.615	98.00	94.83	97.16	0.615
Yes	3.21	2.82	3.37		2.86	5.13		2.00	5.17	2.84	
**Heart disease**											
No	93.57	94.37	93.26	1	94.29	89.74	0.288	92.00	91.38	95.04	0.496
Yes	6.43	5.63	6.74		5.71	10.26		8.00	8.62	4.96	
**Chronic lung disease**											
No	92.77	94.37	92.13	0.787	92.38	94.87	0.747	92.00	89.66	94.33	0.442
Yes	7.23	5.63	7.87		7.62	5.13		8.00	10.34	5.67	
**Chronic kidney disease**											
No	98.80	98.59	98.88	1	98.57	100	1	98.00	100.00	98.58	0.754
Yes	1.20	1.41	1.12		1.43	0.00		2.00	0.00	1.42	
**Immunosuppression**											
No	94.78	98.59	93.26	0.117	94.29	97.44	0.698	92.00	89.66	97.87	0.026
Yes	5.22	1.41	6.74		5.71	2.56		8.00	10.34	2.13	
**Liver pathology**											
No	97.99	98.59	97.75	1	98.57	94.87	0.176	98.00	98.28	97.87	1
Yes	2.01	1.41	2.25		1.43	5.13		2.00	1.72	2.13	
**Cancer**											
No	98.80	98.59	98.88	1	98.57	100.00	1	96.00	100.00	99.29	0.145
Yes	1.20	1.41	1.12		1.43	0.00		4.00	0.00	0.71	
**Pregnancy**											
No			98.31		98.10	100.00	1	98.00	100.00	97.87	0.655
Yes			1.69		1.90	0.00		2.00	0.00	2.13	
**Tobacco consumption (cigarettes/day)**											
0	89.39	90.00	89.14	1	89.37	89.47	0.254	97.96	87.72	87.05	0.172
1–5	4.08	4.29	4.00		3.38	7.89		0.00	7.02	4.32	
> 5	6.53	5.71	6.86		7.25	2.63		2.04	5.26	8.63	
**Physical exercise**											
Inactive	39.36	30.99	42.7	0.114	39.52	38.46	1	46.00	43.10	35.46	0.328
Active	60.64	69.01	57.3		60.48	61.54		54.00	56.90	64.54	
**Risk classification**											
Low	44.58	43.66	44.94	0.768	52.86	0.00	0,001	45.39	36.21	52.00	0.219
Medium	37.75	40.85	36.52		34.76	53.85		40.43	37.93	30.00	
High	17.67	15.49	18.54		12.38	46.15		14.18	25.86	18.00	

Continuous variables: Mean ± SD. Categorical variables: %. *BMI* Body mass index. *WC* waist circumference. Physical exercise: Inactive, 0 times of physical activity performance per week; Active, one or more times of physical activity performance per week. Risk classification: Low, No associated risk factors; Medium, 1 associated risk factor; High, More than 1 associated risk factor. Significance level after correction for multiple comparisons p ≤ 0.05.

### Associations with genetic variants related to metabolism, obesity, inflammation, and chronobiology on SARS-CoV-2 infection

**Figure 1:**
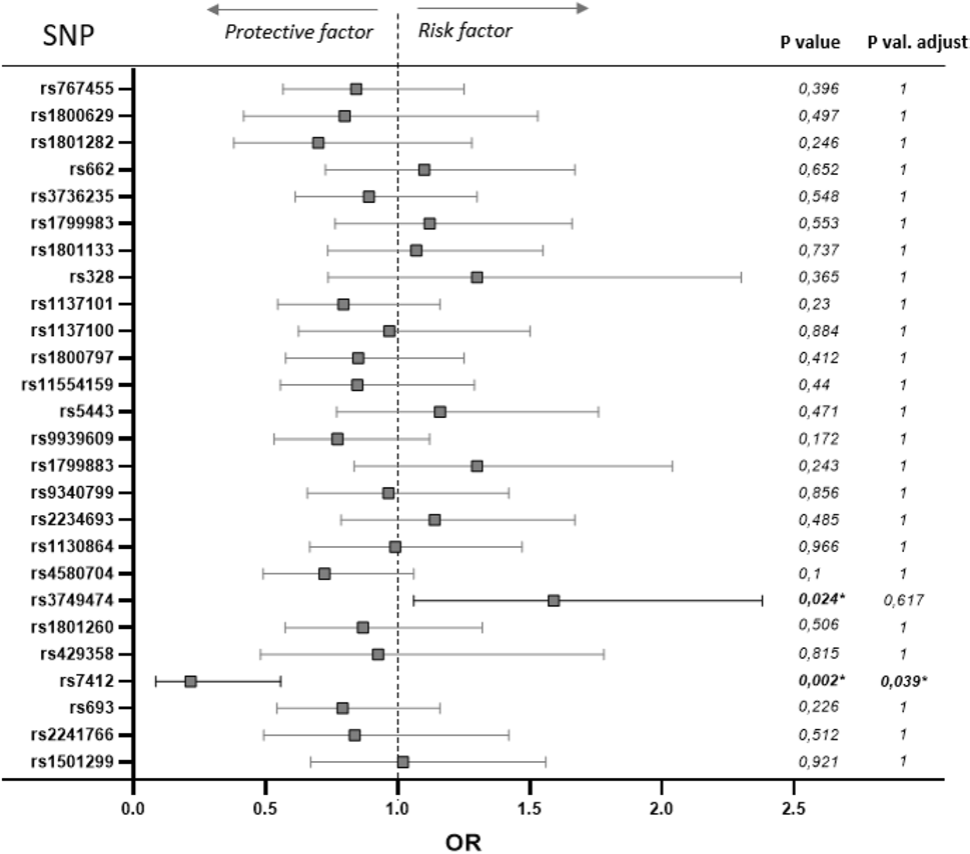
Odds ratio (OR) representing the risk of COVID-19 infection associated to genetic variability of 26 single nucleotide polymorphisms (SNPs) analyzed in 249 volunteers. Confidence interval (CI): 95%. Associations adjusted by sex, age, risk factors, risk of virus exposition, hypertension, symptoms severity and smoking status. The *p*-values were calculated for an additive model and corrected by multiple SNP comparisons by the Bonferroni test.

The objective of this study was to identify putative associations between SARS-CoV-2 infection and genetic factors involved in metabolic or inflammation processes. In this way, 26 SNPs corresponding to genes related to lipid metabolism, obesity, inflammation, and biorhythms were tested for their association with SARS-CoV-2 infection and severity. The frequencies obtained were similar to the European frequencies and the Hardy Weinberg equilibrium was calculated as shown in Table S2. Ordinal logistic regression models were derived for each SNP, adjusted by sex, age, risk factors, risk of virus exposition, hypertension, symptoms severity, and smoking status. An additive model was adopted for the SNPs, where the effect of a homozygotic minor allele genotype was twice the effect of the heterozygotic allele genotype. As predicted endpoint, the three-levels infection status were used (“not infected” *vs* “not clear” *vs* “infected”). Figure 1 displays the odds-ratios and *p* values (before and after Bonferroni correction for multiple tests) obtained for each of the SNPs in these models.

### APOE rs7412 and APOE2 involvement in SARS-CoV-2 infection

A significant association between the genetic variant *APOE* rs7412 and SARS-CoV-2 infection status was found after adjusting for multiple testing corrections. For the additive model, a higher protective effect for each T allele was found with respect to SARS-CoV-2 infection (OR: 0.22; CI: 0.084, 0.557; p = 0.039), which is present in the minor ɛ2 allele of *APOE*. Also, for the dominant model (CC vs CT + TT) the higher protective effect for the T allele (OR: 0.213; CI: 0.081, 0.561; p = 0.046) was found. These results are presented in Table 2. Bootstrap statistics are displayed in Table S3, which shows a small optimism correction and good calibration, signatures of good external predictive power of the model.

**Table 2:** Association of SARS-CoV-2 Infection and *APOE* rs7412 genotype according to genetic models.

Model		No infection (n = 141)	Not clear (n = 58)	Infection (n = 50)	OR (CI)	*p* value	*p* value adjust^a^
**Additive**	CC	118 (83.7)	53 (91.4)	47 (94)	0.216 (0.084, 0.557)	0.002	0.039
	CT	22 (15.6)	5 (8.6)	3 (6)			
	TT	1 (0.7)	0 (0)	0 (0)			
**Dominant**	CC	118 (83.7)	53 (91.4)	47 (94)	0.213 (0.081, 0.561)	0.002	0.046
	CT + TT	23 (16.3)	5 (8.6)	3 (6)			
**Codominant**	CC	118 (83.7)	53 (91.4)	47 (94)	0.227 (0.086, 0.602)	0.011	0.289
	CT	22 (15.6)	5 (8.6)	3 (6)			
	TT	1 (0.7)	0 (0)	0 (0)			

^a^Adjusted by sex, age, risk factors, risk of virus exposure, severity, tobacco consumption and arterial hypertension; Corrected by multiple SNP comparisons (26) by Bonferroni test.

**Figure 2:**
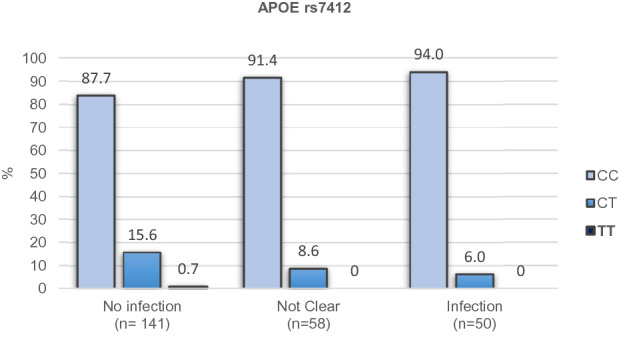
Percentage of the population classified according to SARS-CoV-2 infection status and the *APOE* rs7412 genotype in the additive model. p value = 0.039 adjusted by Bonferroni multiple corrections.

Figure 2 describes the distribution of rs7412 genotypes vs infection status. rs7412 has a T minor allele and a C major allele; in our sample, the CC, CT, and TT genotypes were present in a total of 218, 30 and 1 individuals, respectively. As observed in Fig. 2, for the additive model, when moving from the “not infected” to the “not clear” to the “infected” group, there is a proportional enrichment of the CC genotype in the genetic composition of the corresponding individuals, while the TT genotype is only present in the “not infected” group; the heterozygote also shows a decrease in its contribution upon higher levels of infection, but in a lower proportion.

*APOE* gene has three main alleles, ɛ2, ɛ3 and ɛ4, coding for three corresponding isoforms of apolipoprotein E: E2, E3 and e4, respectively. Table S4 displays the distribution of genotypes in our samples. The main isoform E3 and the minor isoform E4 of ApoE protein have strong affinity to Low Density Lipoprotein Receptor (LDLR) and have been associated to a higher risk of Alzheimer’s disease. On the contrary, E2 isoform has been described as neuroprotective, and presents low affinity for the LDL receptor. In terms of sequences, these isoforms are defined by the amino acids occurring at positions 112 and 158, and determined by rs429358 together with rs7412: E2 isoform: Cys112 (rs429358-T), Cys158 (rs7412-T); E3 isoform: Cys112 (rs429358-T), Arg158 (rs7412-C) and E4 isoform has Arg112 (rs429358-C), Arg158 (rs7412-C). Thus, to determine a putative association between APOE isoform and SARS-CoV-2infection, we next evaluated the combination of the two SNPs rs7412 and rs429358 in the risk of infection by mean of an ordinal logistic model with the same covariables as before, by using a model that included two variables representing the number of ε2 and ε4 alleles, respectively, in the genotype. Table 3 describes the distribution of the three alleles vs infection classes, together with the statistics of the resulting model where a protective effect was found only for ɛ2 allele (OR: 0.207; CI: 0.0796, 0.538; p = 0.001). No significant association was found for ɛ4 (OR: 0.784; 0.402, 1.53; p = 0.476). On the basis of these results, ɛ2 allele seems to be a favorable marker against SARS-CoV-2 infection.

**Table 3:** Association of SARS-CoV-2 Infection and APOE alleles according to additive allelic models.

Allele	No. copies	No infection (n = 141)	Not clear (n = 58)	Infection (n = 50)	OR (CI)	*p* value	*p* value adjust^a^
**ε2**	0	118(83.69)	53(91.38)	47(94)	0.207(0.0796,0.538)	0.001	0.031
	1	22(15.6)	5(8.62)	3(6)			
	2	1(0.71)	0(0)	0(0)			
**ε4**	0	120(85.11)	53(91.38)	40(80)	0.784(0.402,1.53)	0.67	1
	1	18(12.77)	5(8.62)	9(18)			
	2	3(2.13)	0(0)	1(2)			
**ε3**	0	5(3.55)	0(0)	1(2)	Not in model^b^		
	1	38(26.95)	10(17.24)	12(24)			
	2	98(69.5)	48(82.76)	37(74)			

Adjusted by sex, age, risk factors, virus exposure, severity, tobacco consumption, and arterial hypertension.

^a^Corrected by multiple comparisons by Bonferroni test.

^b^This allele is not included as it would result in perfect collinearity.

### T allele of CLOCK rs3749474 might be involved in a potential increased risk of SARS-CoV-2 infection

**Figure 3:**
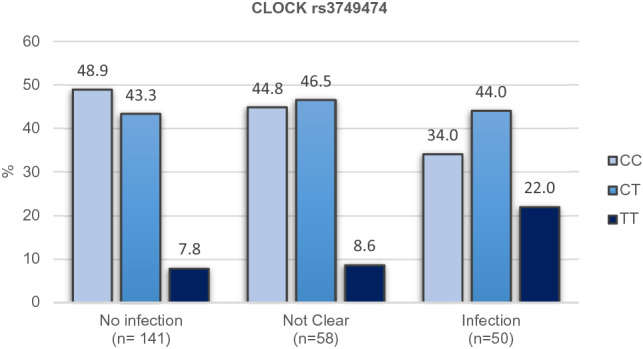
Percentage of the population classified according to SARS-CoV-2 infection status and the *CLOCK* rs3749474 genotype in additive model.

In addition to the association observed with *APOE* genetic variant, the analysis of the genotypes performed showed a positive association between the risk of SARS-CoV-2 infection and *CLOCK* gene rs3749474 polymorphism (p = 0.024), although statistical significance was lost after Bonferroni correction (Fig. 1). This genetic variant has been associated with modulation of metabolic traits, such as energy intake^16^, obesity^17^, and biorhythms^18,19^, and it has also been related to variations in the levels of several cytokines^16^. The best fit was obtained with the additive model, in which each copy of the minor allele T was associated with an increased risk of COVID-19 disease (OR = 1.59, CI: 1.06–2.38) (Table 4). The genotype frequencies obtained from this polymorphism were similar to those described for the European population when analyzing the study population as a whole. However, when stratifying for positive infection, it was observed that among the participants infected by SARS-CoV-2, the TT genotype was present in 22% of the participants, meanwhile only 7.8% of this genotype was present in the group of uninfected subjects (Fig. 3).

**Table 4:** Association of SARS-CoV-2 Infection and *CLOCK* rs3749474 genotype according to genetic models.

	No infection (n = 141)	Not clear (n = 58)	Infection (n = 50)	OR (CI)	*p* value	*p* value adjust^a^
**Additive**						
CC	69 (48.9)	26 (44.8)	17 (34)	1.59 (1.06, 2.38)	0.024	0.617
CT	61 (43.3)	27 (46.6)	22 (44)			
TT	11 (7.8)	5 (8.6)	11 (22)			
**Dominant**						
CC	69 (48.9)	26 (44.8)	17 (34)	1.59 (0.932, 2.7)	0.089	1
CT + TT	72 (51.1)	32 (55.2)	33 (66)			
**Codominant**						
CC	69 (48.9)	26 (44.8)	17 (34)	1.28 (0.724, 2.26)	0.041	1
CT	61 (43.3)	27 (46.6)	22 (44)			
TT	11 (7.8)	5 (8.6)	11 (22)			

^a^Adjusted by sex, age, risk factors, risk of virus exposure, severity, tobacco consumption and arterial hypertension; Corrected by multiple SNP comparisons (26) by Bonferroni test.

In view of these results, an interaction model between *APOE* rs7412 and *CLOCK* rs3749474 variants was performed. We noted that the combination of the two polymorphisms together significantly enhanced the predictive effect that *APOE* variant on the risk of SARS-CoV-2 infection compared to the individual analysis (*p* = 0.02), suggesting a cooperative role of both genetic variants in relation to the infectivity of the virus.

This remarkable finding could be an initial indicator of the potential predictive value of polymorphic variants in the *CLOCK* gene on SARS-CoV2 infection, as well as the correlation that the two SNPs, *APOE* and *CLOCK*, have together regarding susceptibility to COVID-19 infection.

Finally, we investigated the possible modulating effects of diet or medication on SARS-CoV-2 infection. Healthy eating index (IAS), energy intake, total lipids and carbohydrates, simple sugars and saturated fatty acids consumption were tested, as well as consumption of anti-inflammatory, hypotensive or lipid lowering drugs for medication. No significant correlation with SARS-CoV-2 infection or symptoms severity was found in any case.

## Discussion

The present work has focused on the study of genetic factors involved in SARS-CoV-2 infection and its severity in order to broaden the knowledge of the genetic influence on the probability of infection. To this end, single nucleotide genetic variants in genes related to lipid metabolism, inflammation and biorhythms have been analyzed.

A statistically significant association between the genetic variant *APOE* rs7412 and the SARS-CoV-2 infection status was detected for the additive and dominant models, that remained significant after Bonferroni multiple-test correction. Both models suggest that the minor T allele (dominant model) or each copy of the T allele (additive model), would elicits a protective effect against SARS-CoV-2 infection. Nonetheless, given the cross-sectional nature of this study, it is not possible to fully demonstrate from here a causal involvement of the ε2 allele in the observed decrease in infection. However, the significant association reported in this work opens the door to future controlled longitudinal trials capable of confirming this hypothesis, of great interest given the need to understand the risk factors of COVID-19 disease.

This protective effect may be related to multiple processes where apolipoprotein E is implicated including cardiovascular function, immune system and inflammation^20,21^. Previous studies have evidenced the association of *APOE* alleles with susceptibility to infection by viruses, with elevated levels of lipoprotein(a) (Lp(a)), with local extracellular vesicle biogenesis in the brain, with the levels of the Willebrand factor and the modulation of angiotensin converting enzyme 2 (ACE2)^20,22–27^. These processes may be related to a prothrombotic condition which, together with the inflammatory character of SARS-CoV-2 infection, may lead to a higher infection capacity and severity. In addition, different isoforms of apolipoprotein E can give rise to multiple phenotypes, with numerous factors intervening in their biogenesis and expression regulation. For this reason, the multifaceted associations to *APOE* gene require multiple approaches to be taken into account^20^.

The knowledge provided in the current literature regarding COVID-19, refers mainly to *APOE* alleles (ɛ2, ɛ3, and ɛ4) in the population due to the multiallelic nature of apolipoprotein E gene. Nonetheless, the results of the present study revealed a protective effect of the minor T allele of the SNP rs7412 which is found only in the minor ɛ2 allele of *APOE*. Consequently, these results are complementary to those observed in other studies, where the ɛ4 allele increases the risk of SARS-CoV-2 infection and severity of COVID-19 the symptoms^27–30^.

In addition, genome wide significant associations studies (GWAS) related to rs7412 (minor T allele) and higher odds of surviving and longevity have been found^31,32^. On one hand, studies have shown that the rs7412 genetic variant had the strongest association with LDL-TG and RLP-C as well as a significant association with increased TG and HDL-C levels, and with decreased LDL-TG, LDL-C, total cholesterol, non-HDL-C, and Lp(a) levels^33^. In addition, several studies have reported a significant association between the *APOE2*-determining allele of rs7412 and Lp(a) concentrations^34,35^. These investigations similarly revealed a decrease in Lp(a) in the population carrying the allele associated with rs7412^34,35^. In the same time, elevated levels of this lipoprotein have been proposed to be associated with the risk of SARS-CoV-2 infection and with a very high risk of thrombosis^23^. Therefore, these mechanisms could help to explain the potential influence of this genetic variant on SARS-CoV-2 infection. For this reason, this study may help to provide new avenues for studying ApoE purposes, evaluating not only the effect exerted by ɛ4 but also the protective effect of rs7421 and ɛ2 allele against COVID-19.

Subsequently, the effect of the *APOE* gene alleles and the risk of SARS-CoV-2 infection was assessed. As alleles ɛ2, ɛ3 and ɛ4 in *APOE* gene correspond to genotypes rs7412-T/rs429358-T; rs7412-C/rs429358-T; and rs7412-C/rs429358-C, respectively, we evaluated the combination of the two SNPs rs7412 and rs429358 in the risk of infection. In the resulting model, a protective effect was found only for ɛ2 allele after adjusting for the Bonferroni correction. These results, consequently, are in accordance with our hypothesis on the protective effect exerted by the ɛ2 allele. In the same time, published literature reports the beneficial character of apolipoprotein E2 according to cardiovascular diseases, neuroprotection, and other age-related disease traits and survival implicated in the promotion of longevity^27,36,37^. This way, a lower affinity for the LDL receptor, reduced hypertension and/or increased neuroprotective capacity are proposed mechanisms which may contribute to the protective mechanism against SARS-CoV-2 infection^38,39^. Therefore, the multifaceted nature of *APOE* pleomorphic gene, specifically the ɛ2 allele, may have a protective effect against COVID-19 disease.

Besides that, as COVID-19 pandemic evolves, several discouraging long-term effects are being observed. These include neurological manifestations such as smell/taste disorders and non-specific and possibly systemic neuropathological symptoms^40^. This seems to be due to the neurotropic character of the virus, where potential entry of the virus into the vascular endothelial cells of the brain is possible^40,41^. In this scenario, a possible neuroprotective effect could be also hypothesized by the rs7412 or the ɛ2 allele, given its beneficial character in relation to neurodegenerative mechanisms^41^.

Diet and medication do not seem to interfere with infection status and rs7412. However, more studies should be conducted in this direction. This study has been performed in a mainly non-pathological population and a larger sample size may be necessary for future analysis.

The immuno-inflammatory activation as a consequence of a viral infection is a complex system which is affected by multiple factors, including the host circadian clock^14,42^. Disruptions in circadian rhythms have been reported to affect both viral replication and dissemination within the host, as well as innate and adaptive immune responses, which can modulate the infective capacity of the virus and its clinical manifestations^13,14,42,43^. Regarding the infection by SARS-CoV-2, in addition to the previously mentioned, it has been reported that the circadian clock machinery is closely involved in the transcriptional expression of ACE2, the main responsible receptor for the viral entry of SARS-CoV-2 into the cells^44^. In view of the previous evidence, several authors accentuated the urgent need to study the potential contribution of circadian rhythms on COVID-19 disease to clarify whether this relationship can play a role in the spread of this pandemic^45–47^. In this sense, our study shows a positive association between the minor T allele of the rs3749474 variant of the *CLOCK* gene (a core component of the circadian clock) and the risk of infection by SARS-CoV-2 (p = 0.024), although this significance was lost after adjusting for Bonferroni correction, probably due to the low sample size. Despite the fact that the genotype frequencies of this polymorphism were similar to those described in the European population, infected subjects presented an increased incidence of TT + CT genotypes compared to non-infected subjects, pointing to a higher infection rate in the T allele carriers of this polymorphism. This experimental finding describes for the first time a potential direct relationship between a gene involved in the circadian clock and the modulation of SARS-CoV-2 infection, thus opening the door to future studies in this field.

The rs3749474 polymorphism of the *CLOCK* gene has also been associated with altered metabolic features. Subjects who carry the T allele of this variant show a higher energy intake^16^ BMI and risk of obesity^17,48^. After numerous clinical reports, it is a statement that obese patients, and especially those with a higher BMI, are severely affected by COVID-19 and present a worse outcome than patients with lower BMI^49–51^. Again, this supports the link described in our study between the rs3749474 polymorphism and SARS-CoV-2 infection.

It is noteworthy that of all the polymorphisms studied in our population, *APOE* and *CLOCK* polymorphisms have shown significant associations with SARS-CoV-2 infection, and their statistical significance is also enhanced when they are analyzed together in the same model. This suggests a cooperative role of both genes related to the infectivity of this virus. Other authors have previously described cases where the *CLOCK* gene and *APOE* genotypes can interact together modulating the progression of inflammatory-based diseases^52,53^. This reinforces the cooperative role proposed for these polymorphisms on the susceptibility to infection by SARS-CoV-2, as well as the progression and severity of other inflammatory-based diseases in which these genes may play a major role.

The relationship between the severity of the symptoms in the group of individuals infected by SARS-CoV-2 and the analyzed genetic polymorphisms was also assessed. However, no significance was noticed for any of the SNPs tested. These results could suggest that *APOE* and CLOCK could be involved in modulating the risk of SARS-CoV-2 infection as reported, but they may not have a major influence on the progress of the disease once established. However, our study has some limitations. In the first place, the sample size of the infected population might not be large enough to appreciate a significant influence of *APOE* and *CLOCK* in the severity of symptoms. Therefore, it would be advisable to test this association in a large number of infected subjects to ensure if these factors could affect the severity of symptoms caused by COVID-19 disease. Eventually, because of the complicated epidemiological situation Spain was suffering while the study was being performed, the limited availability of diagnostic tests, and the retrospective nature of the study, it was not possible to verify firmly the participants’ SARS-CoV-2 infection. Nonetheless, a validation and verification mechanism of past infection would have been interesting and will be considered in new investigations. Future studies with larger sample size are warranted to contrast the results obtained in this research. Additionally, analyzing these associations in an ethnically diverse population could be relevant as ethnicity has an impact on these variants.

To conclude, although more studies need to be conducted to corroborate the current results, we observed than the minority allele (T) of the genetic variant *APOE* rs7412 and the *APOɛ2* allele, may confer a certain protection against SARS-CoV-2 infection. This observation will allow the performance of fully longitudinal studies to confirm this novel hypothesis from a causative point of view. In addition, the data here obtained and publicly shared will allow the estimation of appropriate sample sizes and power for these future trials.

## Materials and methods

### Subjects and study protocol

Subjects that participated in this study were recruited through the Platform for Clinical Trials in Nutrition and Health (GENYAL), at IMDEA Food Institute (Madrid, Spain). Given the novelty of the COVID pandemic, no information about the variability of the data to collect was available, precluding the use of sample size calculations, so we used the largest sample available. Volunteers included in the GENYAL database were contacted to participate in this study. A total of 371 volunteers of European ancestry were recruited with the objective to link data related to genetic variants, anthropometric measurements, dietary patterns and lifestyle with SARS-CoV-2 infection and severity of COVID-19 disease. Inclusion criteria were the following: previous participation in GENYAL Platform studies, adequate level to understand the research propose, agreement to voluntarily participate in the study and provide informed consent. Only those who refused to give their consent to participate in this study were excluded. CONSORT flow diagram is shown in Fig. S1. Although it could have been better to start with a larger sample, we decided to carry on with the sample available at our hands, on the interest on finding novel associations and risk factors for this novel COVID19 pandemic, and using our resources and experience in the nutrigenomics area.

This study was performed according to the guidelines present in the Declaration of Helsinki and all procedures involving human subjects were approved by the Research Ethics Committee of the IMDEA Food Foundation (CEI 27-666). Written informed consent to enroll in the study was obtained from all the participants. The trial registration number from ClinicalTrials.gov is NCT04067921.

### Anthropometry and lifestyle parameters

Anthropometric measurements such as height, weight and waist circumference were measured following standard validated techniques^54,55^. Body weight was assessed using the body composition monitor BF511 (Omron Healthcare UK Ltd., Kyoto, Japan). Height was assessed using a stadiometer (Leicester-Biological Medical Technology SL, Barcelona). Waist circumferences were measured using a Seca 201 non-elastic tape (Quirumed, Valencia, Spain). The dietary patterns of each participant were assessed using a validated 72-h dietary food record and a food frequency questionnaire^56^. Energy intake, healthy eating index, macro and micronutrients from the dietary records were analyzed using the DIAL software (2.16 version Alce Ingeniería, Madrid, Spain). Regarding lifestyle data, physical activity parameters (Inactive: 0 times of physical activity performance per week/Active: one or more times of physical activity performance per week) and smoking habits (cigarettes/day) were collected from every participants^57^.

### DNA extraction and genotyping

Single nucleotide polymorphisms related to lipid metabolism, obesity, circadian rhythm, immune system, and estrogens were previously screened and selected according to previously published procedures^58,59^. Blood samples were obtained from study participants and stored at − 80 °C until DNA extraction. Genomic DNA from each participant was isolated using the QIAamp DNA Blood Mini Kit (Qiagen Sciences, Inc, Germantown, MD, USA). DNA concentration and quality were assured in a nanodrop ND-2000 spectrophotometer (ThermoScientific, Waltham, MA, USA). Gene genotyping was performed using TaqMan OpenArray plates with the QuantStudio™ 12 K Flex Real-Time PCR System (Life Technologies Inc., Carlsbad, CA, USA) according to the manufacturer’s instructions^60^. The results obtained were analyzed with the TaqMan Genotyper software^61^. Duplicates of the samples were made, obtaining more than 99% of technically valid genotypes.

### COVID-19 data collection

The participants answered a detailed survey of 24 questions related to COVID-19 disease. Questions were stratified into 4 different sections designed to determine: 1—the degree of exposure to SARS-CoV-2, 2—the diagnosis of the disease, 3—symptomatology and severity of the symptoms and 4—associated risk factors. The responses to each of these sections were analyzed and categorized in different clusters according to the following criteria:

1. *Degree of exposure to SARS-CoV-2* Low (Constant home confinement + No contact with people infected by COVID-19), Medium (Rest of participants), High (Coexistence with a person infected by COVID-19/No adherence to COVID-19 security guidelines/Participants with possible exposure to COVID-19 at work).
2. *Diagnosis of the disease* Not infected (Negative PCR/Negative serological test/No perception of symptoms or disease), Not clear (Possible infection but no diagnostic test was performed), Infected by SARS-CoV-2 (Positive PCR / Positive serological test/Typical COVID-19 symptomatology / Diagnosis by medical presumption).
3. *Symptomatology and severity (Table **5**)* Null (No symptoms/Symptoms described by participant as chronic and not associated with COVID-19), Mild-moderate (Mild and moderate symptoms 1–3 weeks/Mild symptoms > 3 weeks/Severe symptoms ≤ 1 week), Severe (Hospitalization period/Pneumonia caused by COVID19/Moderate or severe symptoms > 3 weeks/Severe symptoms 1–3 weeks).
4. *Associated risk factors* Low (0 risk groups), Medium (1 risk group), High (> 1 risk group). Risk groups associated with COVID-19 described: hypertension, diabetes, heart disease, chronic lung disease, chronic kidney disease, immunosuppression, liver pathology, cancer and pregnancy.

**Table 5:** COVID-19 symptomatology used to define severity of disease.

Null	Mild	Moderate	Severe
No symptoms	Mild/moderate symptoms of ≤ 1 week duration	Mild/moderate symptoms for 1–3 weeks	Hospitalization
		Severe symptoms for ≤ 1 week	Pneumonia
	Mild symptoms for 1–3 weeks	Mild symptoms for > 3 weeks	Moderate/severe symptoms for > 3 weeks
			Severe symptoms lasting 1–3 weeks

*Symptoms* Cough, disnea, expectoration (lower respiratory tract secretions), rhinorrhoea, sore throat, chills, fever (mild: ≥ 37.5–< 38 °C; moderate: ≥ 38–< 39 °C ; severe: ≥ 39 °C), diarrhea (mild: < 4 depositions/day; moderate: 4–7 depositions/day; severe: > 7 depositions/day), nausea, vomiting, headache, dizziness, fatigue and myalgia, anosmia, dysgeusia, musculoskeletal pain, loss of appetite, sadness or distress.

### Statistical analysis

The R software, version 3.6.1, was used for all the statistical analyses^62^. A significance level of 0.05 was used in bilateral tests. Missing data was multiple imputed using the mice package, based on conditional chained equations, and using the default imputation methods, namely predictive mean matching for numeric variables, logistic regression for binary variables, polytomous regression for categorical data, and proportional odds logistic regression for ordinal variables^63^. Pooled estimates of parameters and 95% confidence intervals, as well as of *p* values were obtained afterwards from the multiple imputations^64^. The rms package was used to derive ordinal logistic models in the proportional-odds version. These models were fitted to predict infection status for the whole dataset, and symptoms severity in the infected stratum of the sample. A wide set of 26 SNPs related to metabolism and nutrition was tested for significance in the prediction of these endpoints, and sex, age, risk, exposition to infection, hypertension, symptoms severity and smoking status were used as adjustment variables. Multiple-test correction of *p-*values was controlled with the Bonferroni method. The model was validated through bootstrap, using 2000 resamples. An additive model was adopted for the SNPs, where the effect of a homozygotic minor allele genotype is twice the effect of the heterozygotic allele genotype. The use of a codominant or a dominant model did not improve the fit in general, so they were not used. The significance of medication and diet variables was tested by including them in the model in the form of an additive term or an interaction with the rs7412 SNP.

## Supplementary Information

Supplementary Information.

## Data Availability

The datasets presented in this article are not readily available because are part of the GENYAL Platform for clinical trials in nutrition and health (https://www.food.imdea.org/services/ Platform-Clinical-Trials-Nutrition-and-Health) database. This is a database that is currently registered as a collection under the Spanish rules which by policy of the Center will be public afterwards once the data of the entire expected population is gathered. Requests to access the datasets should be directed to ana.ramirez@imdea.org.
