# Supplementary material for: Potential protective effect against SARS-CoV-2 infection by APOE rs7412 polymorphism
Source: Sci Rep. 2022 May 4;12:7247. doi: 10.1038/s41598-022-10923-4 (PMC9065660; doi:10.1038/s41598-022-10923-4)
Supplement: Supplementary file 1 — Supplementary Information. [file 41598_2022_10923_MOESM1_ESM.docx]

**Protective effect against SARS-CoV-2 infection by *APOE* rs7412 polymorphism**

Isabel Espinosa-Salinas^1⸸*^, Gonzalo Colmenarejo^1⸸^, Cristina M. Fernández-Díaz^1⸸^, Marta Gómez de Cedrón^1^, J. Alfredo Martinez^1,2,3^, Guillermo Reglero^1,4^ and Ana Ramírez de Molina ^1^*.

1 IMDEA-Food Institute, CEI UAM+CSIC, 28049 Madrid, Spain;

2 Center for Nutrition Research (CIN), Navarra Institute for Health Research (IdiSNA), 31008 Pamplona, Spain

3 Center of Biomedical Research in Physiopathology of Obesity and Nutrition (CIBEROBN), Institute of Health Carlos III, 28029 Madrid, Spain

4 Institute of Food Science Research (CIAL) CEI UAM+CSIC, 28049 Madrid, Spain

^⸸^ These authors contributed equally.

***** Correspondence: [ana.ramirez@imdea.org](mailto:ana.ramirez@imdea.org); [mariaisabel.espinosa@imdea.org](mailto:mariaisabel.espinosa@imdea.org); +34 91 727 81 00.

**SUPPLEMENTARY MATERIAL**

| **Table S1.** Descriptive data of the study population infected by COVID-19 categorized by severity of symptoms. | | | | |
| --- | --- | --- | --- | --- |
| **Variables** | **Null/Chronic  (n=2)** | **Mild/Moderate (n=31)** | **Severe (n=17)** | ***p*** |
| **Age** | 39.0 ± 8.5 | 45.0 ± 10.3 | 45.8 ± 6.5 | 0.501 |
| **BMI (kg/m2)** | 24.55 ± 5.3 | 28.8 ± 5.9 | 27.7 ± 5.8 | 0.554 |
| **Waist circumference (cm)** | 88.0 ± NA | 94.8 ± 16.5 | 95.3 ± 18.7 | 0.920 |
| **Obesity** |  |  |  |  |
| No | 100 | 61.29 | 58.82 | 0.776 |
| Yes | 0 | 38.71 | 41.18 |  |
| **Hypertension** |  |  |  |  |
| No | 100 | 83.87 | 94.12 | 0.531 |
| Yes | 0 | 16.13 | 5.88 |  |
| **Diabetes** |  |  |  |  |
| No | 100 | 96.77 | 100 | 1 |
| Yes | 0 | 3.23 | 0 |  |
| **Heart disease** |  |  |  |  |
| No | 100 | 93.55 | 88.24 | 0.670 |
| Yes | 0 | 6.45 | 11.76 |  |
| **Chronic lung disease** | |  |  |  |
| No | 100 | 93.55 | 88.24 | 0.678 |
| Yes | 0 | 6.45 | 11.76 |  |
| **Chronic kidney disease** | |  |  |  |
| No | 100 | 96.77 | 100 | 1 |
| Yes | 0 | 3.23 | 0 |  |
| **Immunosuppression** | |  |  |  |
| No | 100 | 93.55 | 88.24 | 0.689 |
| Yes | 0 | 6.45 | 11.76 |  |
| **Liver pathology** |  |  |  |  |
| No | 100 | 96.77 | 100 | 1 |
| Yes | 0 | 3.23 | 0 |  |
| **Cancer** |  |  |  |  |
| No | 100 | 93.55 | 100 | 0.582 |
| Yes | 0 | 6.45 | 0 |  |
| **Pregnancy** |  |  |  |  |
| No | 100 | 96.77 | 100 | 1 |
| Yes | 0 | 3.23 | 0 |  |
| **Tobacco (cigarettes/day)** | |  |  |  |
| 0 | 100 | 96.67 | 100 | 1 |
| 1-5 | 0 | 0 | 0 |  |
| >5 | 0 | 3.33 | 0 |  |
| **Physical exercise** | |  |  |  |
| Inactive | 50 | 41.94 | 52.94 | 0.756 |
| Active | 50 | 58.06 | 47.06 |  |
| **Risk classification** | |  |  |  |
| Low | 100 | 51.61 | 47.06 | 0.627 |
| Medium | 0 | 25.81 | 41.18 |  |
| High | 0 | 22.58 | 11.76 |  |
| Continuous variables: Mean ± SD. Categorical variables: %. BMI: Body mass index. Physical exercise: Inactive, 0 times of physical activity performance per week; Active, one or more times of physical activity performance per week. Risk classification: Low, No associated risk factors; Medium, 1 associated risk factor; High, More than 1 associated risk factor. Significance level p ≤ 0.05. | | | | |

| **Table S2.** Genetic polymorphisms selected in this study | | | |  |  |
| --- | --- | --- | --- | --- | --- |
| **Associated Pathway** | **Gene Name** | **RefSNPs** | **SO TermS** | **MAF** | **HW** |
| LIPID METABOLISM | *APOB* | rs693 | Synonymous Variant | 0.48 | 0.427 |
|  | *APOE* | rs7412 | Missense Variant | 0.06 | 0.973 |
|  |  | rs429358 | Missense Variant | 0.08 | 0.041 |
|  | *FABP2* | rs1799883 | Missense Variant | 0.24 | 0.202 |
|  | *LPL* | rs328 | Stop Gained | 0.13 | 0.745 |
|  | *MTHFR* | rs1801133 | Missense Variant | 0.41 | 0.207 |
|  | *NOS3* | rs1799983 | Missense Variant | 0.38 | 0.872 |
|  | *OLR1* | rs3736235 | Intron Variant | 0.45 | 0.861 |
|  | *PON1* | rs662 | Missense Variant | 0.28 | 0.990 |
|  | *PPARG* | rs1801282 | Intron Variant | 0.10 | 0.112 |
| OBESITY | *ADIPOQ* | rs1501299 | Intron Variant | 0.26 | 0.847 |
|  |  | rs2241766 | Synonymous Variant | 0.17 | 0.393 |
|  | *FTO* | rs9939609 | Intron Variant | 0.39 | 0.472 |
|  | *GNB* | rs5443 | Non Coding Transcript Variant | 0.35 | 0.055 |
|  | *LEPR* | rs1137100 | Missense Variant | 0.23 | 0.701 |
|  |  | rs1137101 | Missense Variant | 0.41 | 0.876 |
| CIRCADIAN RHYTHM | *CLOCK* | rs1801260 | 3 Prime UTR Variant | 0.27 | 0.898 |
|  | *CLOCK* | rs3749474 | 4 Prime UTR Variant | 0.33 | 0.923 |
|  | *CLOCK* | rs4580704 | Intron Variant | 0.36 | 0.588 |
| IMMUNE SYSTEM | *CRP* | rs1130864 | Intron Variant | 0.30 | 0.209 |
|  | *IFI30* | rs11554159 | Missense Variant | 0.28 | 0.550 |
|  | *IL6* | rs1800797 | Intron Variant | 0.34 | 0.038 |
|  | *TNF* | rs1800629 | Upstream Transcript Variant | 0.11 | 0.552 |
|  | *TNFRSF1A* | rs767455 | 5 Prime UTR Variant | 0.38 | 0.604 |
| OESTROGENS | *ESR1* | rs2234693 | Intron Variant | 0.47 | 0.604 |
|  | *ESR1* | rs9340799 | Intron Variant | 0.40 | 0.795 |
| MAF: Minor Allele Frequency by the Study Population; SO: Sequence Ontology; HW: Hardy-Weinberg Equilibrium. | | | | | |

| **Table S3.** Bootstrap validation of additive model with APOE rs7412 genotype^a^ | | | | | |
| --- | --- | --- | --- | --- | --- |
| **Index** | **index.orig** | **training** | **test** | **optimism** | **index.corrected** |
| Dxy | 0.524 | 0.559 | 0.494 | 0.066 | 0.458 |
| R2 | 0.321 | 0.358 | 0.287 | 0.071 | 0.25 |
| Intercept | 0 | 0 | -0.0351 | 0.0351 | -0.0351 |
| Slope | 1 | 1 | 0.829 | 0.171 | 0.829 |
| ^a^2000 bootstrap resamples used. Dxy = Somers' Dxy rank correlation; R2 = R-squared index; intercept and slope: the intercept and slope of an overall logistic calibration equation. We observe a small optimism correction, together with an intercept near 0 and slope near 1, as expected for a good predictive power. | | | | | |

| **Table S4.** Frequency of the study population based on *APOE* genotypes. | | | |
| --- | --- | --- | --- |
| **Genotypes** | **n (%)** | **Alelles** | **n (%)** |
| ɛ2ɛ2 | 1 (0.4) | ɛ2 | 32 (6.4) |
| ɛ2ɛ3 | 29 (11.6) | ɛ3 | 426 (85.5) |
| ɛ2ɛ4 | 1 (0.4) | ɛ4 | 40 (8.03) |
| ɛ3ɛ3 | 183 (73.5) |  |  |
| ɛ3ɛ4 | 31 (12.5) |  |  |
| ɛ4ɛ4 | 4 (1.6) |  |  |


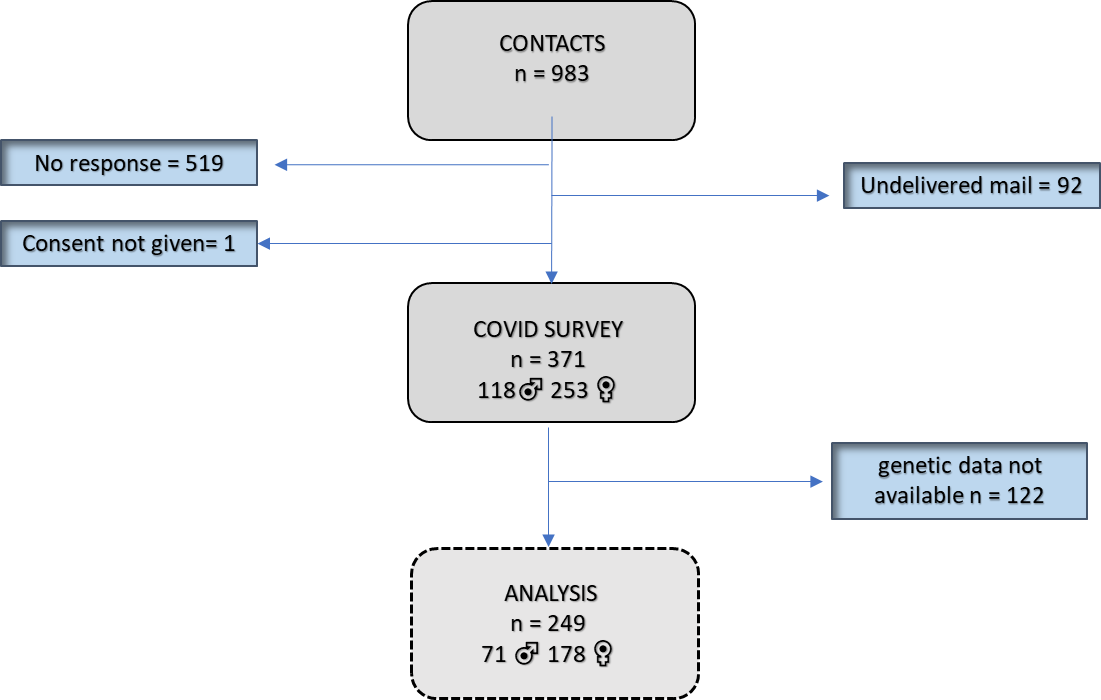


**Figure S1. CONSORT flow diagram.**
